# Ecological Trait-Based Digital Categorization of Microbial Genomes for Denitrification Potential

**DOI:** 10.3390/microorganisms12040791

**Published:** 2024-04-13

**Authors:** Raphael D. Isokpehi, Yungkul Kim, Sarah E. Krejci, Vishwa D. Trivedi

**Affiliations:** Oyster Microbiome Project, College of Science, Engineering and Mathematics, Bethune-Cookman University, Daytona Beach, FL 32114, USA; krejcis@cookman.edu (S.E.K.); trivediv@cookman.edu (V.D.T.)

**Keywords:** archaea, *Arcobacteraceae*, bacteria, bioinformatics, data investigations, denitrification, ecological trait, genomes, microbial ecology, KEGG Orthology, synthetic denitrifying communities, visual analytics

## Abstract

Microorganisms encode proteins that function in the transformations of useful and harmful nitrogenous compounds in the global nitrogen cycle. The major transformations in the nitrogen cycle are nitrogen fixation, nitrification, denitrification, anaerobic ammonium oxidation, and ammonification. The focus of this report is the complex biogeochemical process of denitrification, which, in the complete form, consists of a series of four enzyme-catalyzed reduction reactions that transforms nitrate to nitrogen gas. Denitrification is a microbial strain-level ecological trait (characteristic), and denitrification potential (functional performance) can be inferred from trait rules that rely on the presence or absence of genes for denitrifying enzymes in microbial genomes. Despite the global significance of denitrification and associated large-scale genomic and scholarly data sources, there is lack of datasets and interactive computational tools for investigating microbial genomes according to denitrification trait rules. Therefore, our goal is to categorize archaeal and bacterial genomes by denitrification potential based on denitrification traits defined by rules of enzyme involvement in the denitrification reduction steps. We report the integration of datasets on genome, taxonomic lineage, ecosystem, and denitrifying enzymes to provide data investigations context for the denitrification potential of microbial strains. We constructed an ecosystem and taxonomic annotated denitrification potential dataset of 62,624 microbial genomes (866 archaea and 61,758 bacteria) that encode at least one of the twelve denitrifying enzymes in the four-step canonical denitrification pathway. Our four-digit binary-coding scheme categorized the microbial genomes to one of sixteen denitrification traits including complete denitrification traits assigned to 3280 genomes from 260 bacteria genera. The bacterial strains with complete denitrification potential pattern included *Arcobacteraceae* strains isolated or detected in diverse ecosystems including aquatic, human, plant, and Mollusca (shellfish). The dataset on microbial denitrification potential and associated interactive data investigations tools can serve as research resources for understanding the biochemical, molecular, and physiological aspects of microbial denitrification, among others. The microbial denitrification data resources produced in our research can also be useful for identifying microbial strains for synthetic denitrifying communities.

## 1. Introduction

**Figure A1:**
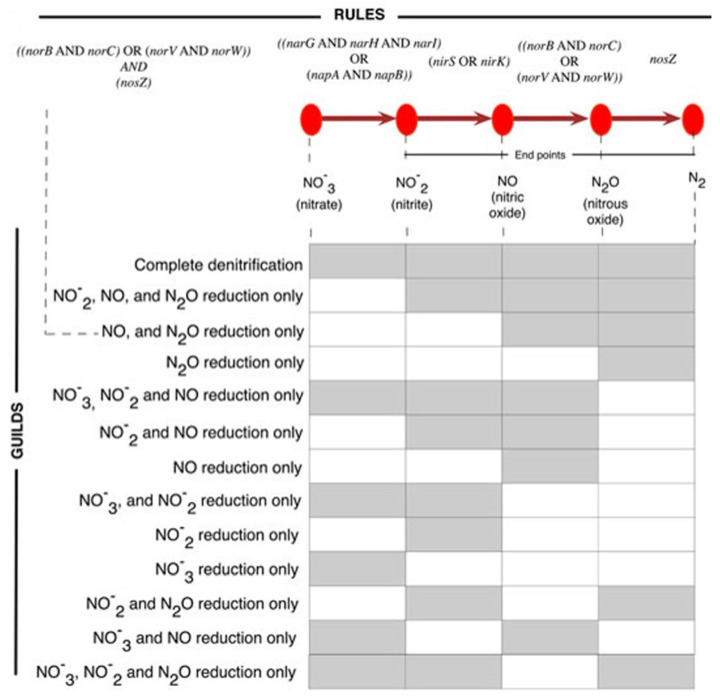
An example of the denitrification trait inferences according to rules of the end products of reduction reactions of nitrate, nitrite, nitric oxide, and nitrous oxide. The presence or absence of protein families (italicized gene symbols) maps to complete and incomplete denitrification traits. The grey and white colors indicate presence and absence respectively of the gene(s) for the denitrification step. The source of the image is an open-access article by Karaoz and Brodie [10].

Microorganisms encode proteins that function in the transformations of useful and harmful nitrogenous compounds in the global nitrogen cycle [1,2]. The major transformations in the nitrogen cycle are nitrogen fixation, nitrification, denitrification, anaerobic ammonium oxidation (anammox), and ammonification [3,4]. Nitrogen cycling is central to ecosystem functioning including by microbial sources and the sink of nitrogenous compounds [5,6,7]. This report focuses on the complex biogeochemical process of denitrification which, in the complete form, consists of a series of four enzyme-catalyzed reduction reactions that transform nitrate to nitrogen gas [4,8]. Enwall et al. [9] described denitrification as “an alternative pathway for microorganisms to respire under oxygen-limited conditions, using nitrogen oxides as electron acceptors”. Denitrification is a strain-level trait, and denitrification potential (functional performance) can be inferred from trait rules that rely on the presence or absence of genes for denitrifying enzymes such a nitrous oxide reductase [10,11]. Digital categorization of biological knowledge (e.g., denitrification) with representations such as trait rules, ontologies, and controlled vocabularies support knowledge sharing and discovery across biological domains [12,13]. Microbial genome web portals provide large-scale taxonomic-strain level datasets that include annotations of enzymes encoded by microbial genomes [14]. For example, the Integrated Microbial Genomes & Microbiomes (IMG/M) system provides tools to retrieve lists of microbial genomes with specific functional annotation entries such as Enzyme Commission (E.C.) number, Clusters of Orthologous Genes (COG), Kyoto Encyclopedia of Genes and Genomes (KEGG) Orthology, and Pfam: protein families and domains [15]. Furthermore, researchers can download datasets of interest such as genomes or genes annotated with specific KEGG or COG denitrification entries [16]. In our prior research [17], we synthesized downloaded datasets of genomes using binary number representations to categorize the genomes. Karaoz and Brodie [10] in a journal article titled “MicroTrait: a toolset for trait-based representation of microbial genomes”, recommended the need for new data synthesis approaches for microbial trait datasets, where data from microbial genomes are at the core of investigating the environmental roles and functional performance of microorganisms. The 16 categories of denitrification potential including complete denitrification were defined by Karaoz and Brodie [10] (Appendix A, Figure A1).

There are protein function annotations from over 100,000 microbial genomes [15,18] and at least 500,000 scholarly publications on denitrification in Google Scholar, including bioinformatics studies and notable reviews [18,19,20,21,22]. These online molecular and scholarly literature resources present opportunities to generate multivariate large-scale datasets that are ready for diverse types of data investigations including data synthesis and data analytics. The 12 genes for enzymes involved in the canonical denitrification pathway are in four groups: (1) nitrate reductases (*narG*, *narH*, *narI*, *napA*, *napB*); (2) nitrite reductase (*nirK*, *nirS*); (3) nitric oxide reductase (*norB*, *norC*, *norV*, *norW*); and (4) nitrous oxide reductase (*nosZ*) [5,10,18,23]. Furthermore, the enzyme complexes are narGHI, napAB, norBC, and norVW. Thus, with the 12 enzymes combined in absence (“0”) or presence (“1”) values, it is possible to assign a microbial strain to 1 of 4096 twelve-digit binary numbers.

A widely used bioinformatics tool for predictive functional profiling of 16S rRNA gene amplicon sequencing from environmental samples is Phylogenetic Investigation of Communities by Reconstruction of Unobserved states (PICRUSt2) [24,25]. PICRUSt2 prediction of function relies on similarities to genome annotations in IMG/M [15]. In addition, studies have used PICRUSt2 to predict nitrogen-cycling pathways of microbial communities in an ecosystem [26,27,28,29]. A limitation of PICRUSt2 is its inability to distinguish strain-level functionality [24,28]. In addition, the ecological trait relevance (e.g., denitrification potential) of the list of KEGG orthologues predicted by PICRUSt2 requires interpretation. Thus, there is an opportunity to provide datasets and interactive data investigation resources that contain strain-level categorization of denitrification potential according to the 16 possible categories of Karaoz and Brodie [10]. Interactive computational resources designed with general purpose software (e.g., spreadsheet and visual analytics) for investigating denitrification potential datasets can be also be useful for the interpretation of denitrification traits predicted by tools such as PICRUSt2, PAPRICA (PAthway PRediction by phylogenetIC plAcement) [30], and Tax4Fun2 [31].

Despite the global significance of denitrification [2,32] and associated large-scale genomic and scholarly data sources, there is lack of datasets and interactive computational tools for investigating microbial genomes according to denitrification trait rules. Therefore, our goal is to categorize archaeal and bacterial genomes by denitrification potential based on denitrification traits defined by rules of enzyme involvement in the denitrification reduction steps [5,10,11]. This goal is important because denitrification is a taxonomic-strain level trait and because the denitrification traits of a newly sequenced microbial genome are useful to designing research on the biochemical, molecular, and physiological aspects of microbial denitrification among others. The abundance of genome sequences of microbial strains has led to a growing interest in synthetic microbial communities (SynComs) or consortia (e.g., synthetic denitrifying communities) for biotechnological, bioengineering, and ecosystem function applications [7,33,34]. A critical initial stage in the design of optimal synthetic microbial communities is identifying microbial strains that constitute the microbial community [35]. Thus, researchers will benefit from datasets and easy-to-use computational tools for identifying strains for the optimal design of a synthetic microbial community. Therefore, the first objective of our microbial denitrification data investigations was to construct a microbial denitrification potential dataset containing archaeal and bacterial genomes annotated with at least one of the twelve KEGG denitrification enzyme entries. The second objective was to develop interactive computational resources with spreadsheet software and visual analytics software to support investigations of the microbial denitrification dataset.

Since marine invertebrates such as clams, mussels, and oysters can be hosts to microorganisms that contribute to denitrification [36,37], we compiled patterns of presence or absence of denitrifying enzyme genes and the denitrification potential for bacteria genera associated with the Eastern oyster (*Crassostrea virginica*). One reason for the interest in the denitrification potential of bacteria associated with the Eastern oyster is that oyster aquaculture is associated with low greenhouse gas emissions [38]. Metagenomics sequencing technologies have aided in the identification of archaea and bacteria associated with oyster anatomical parts including the gills, gut, hemolymph, mantle, pallial fluid, stomach, and shell [37,39,40,41,42,43]. In studies with a focus on the denitrification potential of the oyster microbiome [37,42], the bacteria genera identified include *Clostridium*, *Endozoicomonas*, *Erythrobacter*, *Mycoplasma*, *Neptunibacter*, *Pleurocapsa*, *Psychrobacter*, *Pseudomonas*, *Pseudoalteromonas*, *Shewanella*, *Synechococcus*, and *Vibrio*.

We report an ecosystem and taxonomic annotated dataset of 62,624 microbial genomes (866 archaea and 61,758 bacteria) that encode at least one of the twelve denitrifying enzymes in the four-step canonical denitrification pathway. The bacterial strains with complete denitrification potential pattern included *Arcobacteraceae* strains isolated or detected in diverse ecosystems including aquatic, human, plant, and Mollusca (shellfish). In addition, we developed a set of accessible and easy-to-use data-investigation interfaces (spreadsheet and visual analytics) to support human interaction with the microbial denitrification dataset. The visual analytics interface also includes searches for gene symbols of the denitrification enzymes in scholarly databases. The microbial denitrification data resources can be used for identifying microbial strains for synthetic denitrifying communities (SDCs).

## 2. Materials and Methods

### 2.1. Data Sources for Functional Annotation Identifiers of Denitrification Enzyme Genes

The microTrait framework of the genome-derived functional traits of ecological relevance is the source of the 12 gene symbols for enzymes involved in the four-step canonical denitrification pathway [10]. Additionally, the microTrait framework provides rules that describe the participating enzymes and the products of the reduction reactions (Appendix A, Figure A1). The Kyoto Encyclopedia of Genes and Genomes (KEGG) resource is the source of the functional annotation identifiers for the 12 gene symbols [10,44]. The 12 gene symbols and associated KEGG Orthology Identifiers are *narG* (K00370), *narH* (K00371), *narI* (K00374), *napA* (K02567), *napB* (K02568), *nirK* (K00368), *nirS* (K15864), *norB* (K04561), *norC* (K02305), *norV* (K12264), *norW* (K12265), and *nosZ* (K00376). The KEGG functional annotation is among the pathway annotation databases commonly used in the functional prediction of gene function including denitrification [24,25,30,31,45].

### 2.2. Construction of a Denitrification Potential of Archaeal and Bacterial Genomes Dataset 

**Figure 1:**
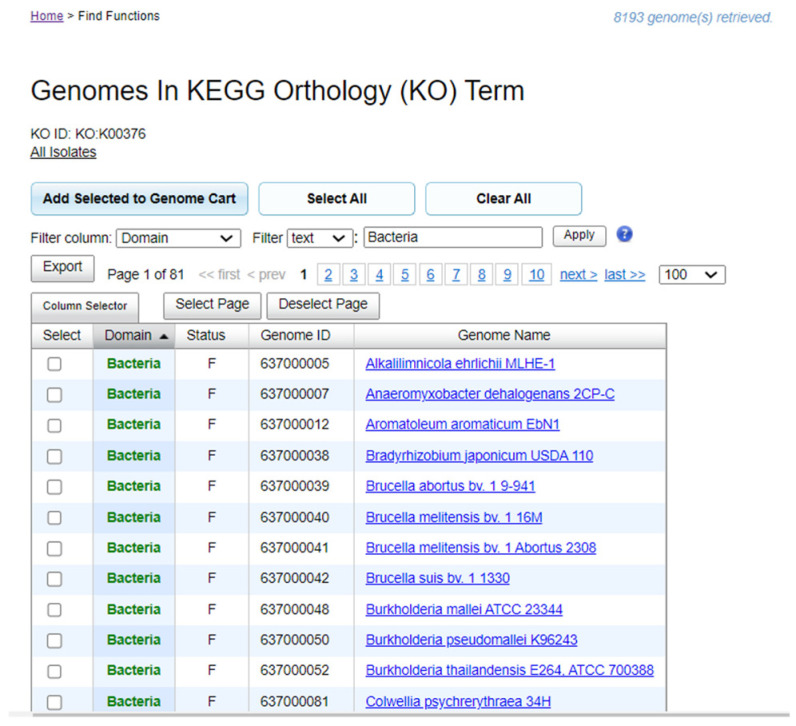
A screenshot of an Integrated Microbial Genomes and Microbiomes (IMG/M) webpage displaying microbial genomes with annotation for a KEGG Orthology (KO) term identifier. The example shown is for nitrous oxide reductase with KO identifier K00376, retrieving 8193 genomes.

The construction of the dataset followed the formats used for trait datasets such as wide table and long table [12]. The archaeal and bacterial genomes (strains) with the genes annotated with each of the 12 KEGG Orthology (KO) terms were retrieved in the Integrated Microbial Genomes and Microbiomes (IMG/M) data management and analysis system [15]. We used the uniform resource locator (URL) script that finds genomes with the denitrification KEGG Orthology (KO) term (for example, K00376 for nitrous-oxide reductase, nosZ). An alternative approach was to use the IMG/M Find Genes interface to retrieve the genomes with the KO term. Each of the retrieved datasets contained columns for domain (taxonomic domain), status (sequencing status), genome ID (identifier assigned by IMG/M), and genome name (Figure 1). As the data-investigation-ready approach for datasets collected - from microbial genome web portals [14], we used binary numbers (0 and 1) of varying length of the binary number to synthesize the availability of data on functional annotations in microbial genomes [17]. We also adapted an approach that use binary numbers to synthesize the direction of gene arrangements assigned to genes in microbial genomes [46].

**Figure 2:**
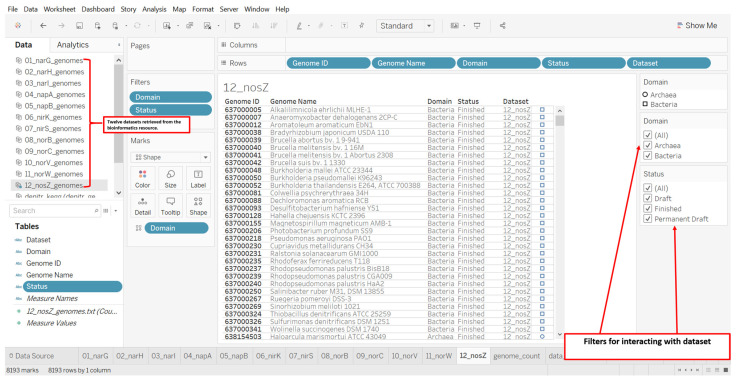
A screenshot of the design of a visual analytics resource for constructing a dataset of microbial genomes from the dataset retrieved from the bioinformatics resource (IMG/M). The example shown is for nitrous oxide reductase with KEGG Orthology identifier K00376. The filters in the design allow for the display of a dataset with options for taxonomic domain and genome sequencing status.

Each dataset was uploaded into Tableau, a visual analytics software [47], and a column “Dataset” with identical value for all the records was added as a calculated field. For example, we added "01_narG” and “12_nosZ” to the genome list for *narG* and nosZ, respectively (Figure 2). We added this additional column to construct the data-investigation-ready dataset consisting of the 12 datasets of genomes. We downloaded the labeled datasets and then combined them in Tableau using the “Append Data from File” feature.

We constructed an integrated dataset from the 12 datasets in the visual analytics software. The columns in the dataset included those for genome ID and genome name; 12 columns for the denitrification KEGG Orthology with entries of “0” (absence of KO in genome) or “1” (presence of KO in genome); and a column that joined all the KO binary digits to form a 12-digit binary number, which we termed “Denitrifying Enzymes Pattern”. The order of the digits in the binary number reflects the enzymes in the four steps of denitrification: (1) nitrate reductases (narG, narH, narI, napA, napB); (2) nitrite reductase (nirK, nirS); (3) nitric oxide reductase (norB, norC, norV, norW); and (4) nitrous oxide reductase (nosZ). Therefore, the 12th digit is for the presence or absence of the gene for nitrous oxide reductase in a microbial genome.

An additional 4-digit binary number (“Denitrification Pattern”) was constructed from the 12-digit “Denitrifying Enzymes Pattern” using the rules for denitrification traits as described by Karaoz and Brodie [10] (Appendix A, Figure A1). For example, complete denitrification trait or potential (denitrification pattern 1111) will involve the presence of the appropriate combinations of enzymes to catalyze each of the four steps of denitrification.

We used the IMG/M Find Genomes tools to retrieve relevant fields to facilitate taxonomic and ecosystem interpretation as well as research advances such as the design of synthetic denitrifying communities using the denitrification potential dataset. The categories of data fields are (1) Genome Database Taxonomy (GTDB) Toolkit (GTDB-Tk Domain, GTDB-Tk Family, GTDB-Tk Genus, GTDB-Tk Order, GTDB-Tk Phylum, GTDB-Tk Species) and (2) ecosystem classes (Ecosystem, Ecosystem Category, Ecosystem Type, Ecosystem Subtype, and Specific Ecosystem). We also derived a column “Genus” from the “Genome Name” column by extracting the text before the space in the “Genome Name” column. For example, “*Pseudomonas aeruginosa* PAO1” would be “*Pseudomonas*”.

### 2.3. Designs and Implementations of Visual Analytics Resources to Support Human Interaction with the Dataset on the Denitrification Potential of Microbial Genomes

We designed spreadsheet and visual analytics worksheets to include filters and other interaction techniques for interaction with the data in the worksheets. The interaction techniques can support the performance of complex cognitive activities, which are information intensive and involve complex human cognition (mental processes) [48,49]. A catalog of 32 interaction techniques that support the performance of complex cognitive activities (such as knowledge discovery, problem-solving, decision-making) [48] guided the designs and implementation of the visual analytics resources (worksheets and dashboards) in Tableau [47]. The overall design of the visual analytics resource for interacting with the dataset is an enclosure table view that groups the genome names according to a 4-digit and a 12-digit binary number. Each row in the view also has a shape mark to indicate the genome sequencing status. We included filtering and searching interaction techniques in our design to help us identify a subset of the dataset to perform complex cognitive activities. In this project, the design of a core visual analytics resource allows for the querying of the dataset using the columns such as those for genome name, genome ID, denitrification pattern, denitrification traits, and denitrifying enzymes pattern. An additional feature of the design is the uniform resource locator (URL) action that provides a hyperlink to a web page of Google Scholar, a web search engine for scholarly literature and academic resources.

### 2.4. Denitrification Potential Categorization of Bacterial Genera Associated with Eastern Oyster (Crassostrea virginica)

Since oysters are filter feeders and since the gills are the filtering tissue in constant contact with the surrounding water [37,50,51], we designed visual analytics worksheets to categorize according to denitrification potential for a set of bacteria genera (*Arcobacter*, *Bradyrhizobium*, *Caulobacter*, *Marinifilum*, *Pelomonas*, *Pseudoalteromonas*, *Pseudomonas*, *Psychrobacter*, and *Sphingomonas*) associated with oyster gills [52].

## 3. Results

### 3.1. Dataset on the Denitrification Potential of Microbial Genomes

The dataset on the denitrification potential of 62,624 microbial genomes (866 archaea and 61,758 bacteria) consisted of 36 variables (columns) from genome annotations and denitrification annotations (Table 1). The Genome ID from the IMG/M system was the unique identifier for each genome. We calculated/derived the denitrification annotations categories (denitrification potential and denitrifying enzymes) from the input datasets retrieved from the IMG/M system (Table 2). In the dataset, the gene for the nitrous oxide reductase (*nosZ*), the enzyme for the last step of denitrification, was present in 181 archaea and 8009 bacteria genomes (Table 2). There were at least 100 archaeal and 2000 bacteria genera as well as 484 twelve-digit denitrification patterns in the dataset. We observed 1021 strains with two, three, four, or five genome sequences. The four strains with five genome sequences in the microbial denitrification potential dataset were the following: (1) *Brucella melitensis* bv. 1 16 M; (2) *Corynebacterium aurimucosum* CN-1, ATCC 700975; (3) *Escherichia coli* EC2; and (4) *Pseudomonas aeruginosa* DSM 50071. The Supplementary Materials and Data Availability sections of this report provide details on how to access the denitrification potential dataset.

**Figure 3:**
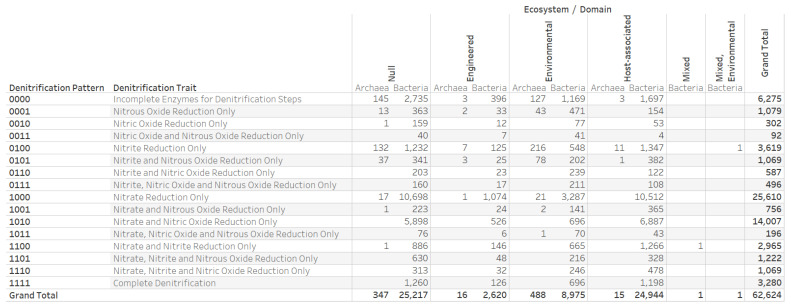
Distribution of denitrification patterns and denitrification traits assigned to a set of 62,624 microbial genomes consisting of 866 archaeal and 61,758 bacterial genomes. “Null” means an absence of annotation.

**Figure 4:**
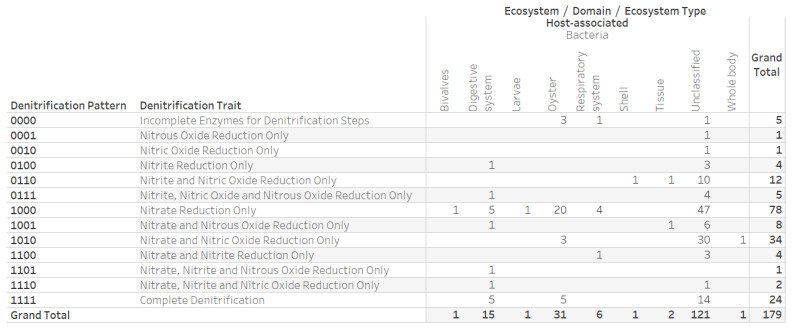
Distribution of denitrification patterns, denitrification traits, and ecosystem types for 179 bacterial genomes annotated with the ecosystem of the host-associated and ecosystem category of Mollusca. The five genomes assigned to the oyster ecosystem type were from four strains of *Roseibium album* (CECT 5094, CECT 5095, CECT 5096, and CECT 7551) and *Ruegeria denitrificans* CECT 5091.

The distribution of the 16 denitrification patterns and associated denitrification traits in ecosystems for archaeal and bacterial genomes revealed the potential for complete denitrification by 3280 bacterial genomes (Figure 3). The denitrification-potential dataset contained five IMG/M ecosystem annotations (engineered, environmental, host-associated, mixed, and mixed, environmental) assigned to 37,407 of the 62,624 genomes. We verified that the 8190 genomes with the nitrous oxide reduction trait (nosZ) were associated with denitrification patterns 0001 (1079 genomes), 0011 (92 genomes), 0101 (1069 genomes), 0111 (496 genomes), 1001 (756 genomes), 1011 (196 genomes), 1101 (1222), and 1111 (3280 genomes). The 179 genomes annotated with the Mollusca ecosystem category included 31 genomes with an ecosystem type annotation of oyster (Figure 4).

### 3.2. Designs and Implementations of Visual Analytics Resources to Support Interaction with the Dataset on the Denitrification Potential of Microbial Genomes

**Figure 5:**
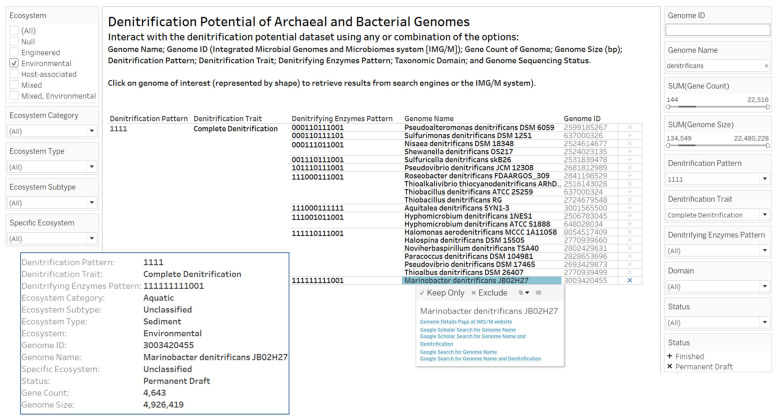
A screenshot of a visual analytics resource to support interaction with the dataset on denitrification potential of archaeal and bacterial genomes with an emphasis on filtering by ecosystem options. The interaction worksheet provides options and links to external resources (IMG/M website, Google Search and Google Scholar). The insert box on the left was obtained from clicking the sequencing status symbol associated with *Marionobacter denitrificans* JB02H27, a bacteria isolated from marine sediment and known to reduce nitrite and nitrate to gaseous nitrogen [54]. The webpage link to the interactive version of the visual analytics resource is available in the Supplementary Materials section.

We designed and implemented several visual analytics worksheets and dashboards to support the performance of investigation, knowledge discovery, decision-making, and other complex cognitive activities on the denitrification potential of microbial genomes dataset. A visual analytics worksheet (Figure 5) design allows for interaction with the denitrification potential dataset using the columns in the genome, ecosystem, and denitrification potential category (Table 1). Based on the taxonomic description in valid publications of microbial strains, microorganisms with “denitrificans”, meaning denitrifying, can provide a subset of genomes with evidence for denitrification enzymes (for example “reduces nitrate to nitrogen” as in the description of *Sulfuricella denitrificans* skB26 [53]). The constructed dataset contains 2 archaea and 116 bacteria genomes with the genome name containing “denitrificans” (meaning denitrifying) assigned to 13 denitrification traits. As shown in Figure 5, 20 genome names were displayed when the interaction filters were (1) environmental ecosystem, (2) a genome name that contained “denitrificans”, and (3) a denitrification pattern for complete denitrification of “1111”. The “Denitrifying Enzymes Pattern” for *Marinobacter denitrificans* JB02H27 [54] of “111111111001” lacks the genes for anaerobic nitric oxide reductase flavorubredoxin (*norV*) and nitric oxide reductase FlRd-NAD(+) reductase (*norW*). Other genera that have species with “denitrificans” in species name are *Aquitalea*, *Halomonas*, *Halospina*, *Hyphomicrobium*, *Nisaea*, *Noviherbaspirillum*, *Paracoccus*, *Pseudoalteromonas*, *Pseudovibrio*, *Roseobacter*, *Shewanella*, *Sulfuricella*, *Thioalkalivibrio*, *Thioalbus*, and *Thiobacillus*. We observed shared 12-digit presence or absence patterns of denitrifying enzymes by genomes. For example, the digital categorization process assigned pattern “000111011001” to genomes of *Nisaea denitrificans* DSM 18348 and *Shewanella denitrificans* OS217 (Figure 5).

**Figure 6:**
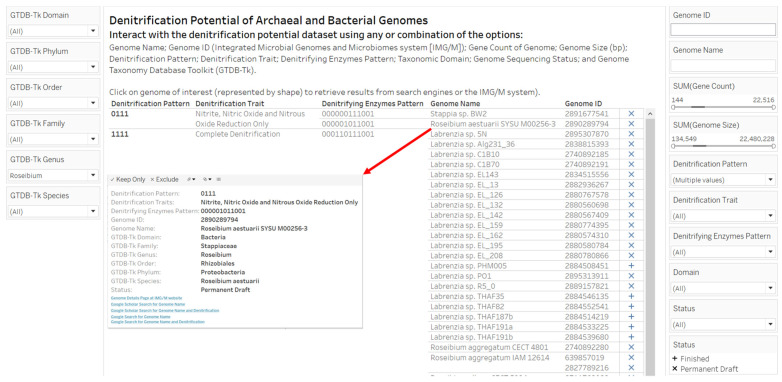
A screenshot of a visual analytics resource to support human interaction with the dataset on denitrification potential of archaeal and bacterial genomes with emphasis on filtering by taxonomic options. The interaction worksheet provides options as well as connection to external resources (IMG/M website, Google Search and Google Scholar). The insert image with GTDB-Tk taxonomic assignments was obtained by clicking the sequencing status symbol associated with *Roseibium aestuarii* SYSU M00256-3, a bacteria isolated from an estuary and known to be unable to reduce nitrate [55]. The webpage link to the interactive version of the visual analytics resource is available in the Supplementary Materials section.

Another visual analytics design emphasized the filtering of the dataset by taxonomic classifications. In Figure 6, the view displayed is for filtering the dataset by *Roseibium* GTDB-Tk Genus. We filtered the dataset by *Roseibium* as we observed the annotation of *Roseibium* genomes with oyster host-associated ecosystem (Figure 4). The view produced by the interaction contains three Denitrifying Enzymes Patterns (000000111001, 000001011001 and 000110111001), and two types of Denitrification Traits: (1) Nitrite, Nitric Oxide and Nitrous Oxide Reduction only and (2) Complete Denitrification.

### 3.3. Denitrification Potential Categorization of Bacterial Genera Associated with Eastern Oyster (Crassostrea virginica)

**Figure 7:**
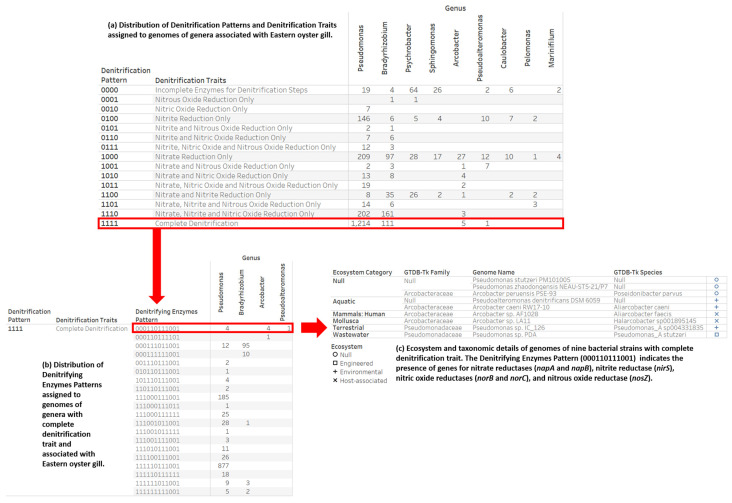
Three stages of interactive data investigation for the denitrification potential of bacterial genera associated with the Eastern oyster (*Crassostrea virginica*). We obtained the list of nine genera from the study of bacteria associated with the gill tissues of the Pacific oyster (*Crassostrea gigas*) and Eastern oyster [52].

The nine bacteria genera associated with the gill tissue of the Eastern oyster whose strains were categorized by patterns of denitrification potential are *Arcobacter*, *Bradyrhizobium*, *Caulobacter*, *Marinifilum*, *Pelomonas*, *Pseudoalteromonas*, *Pseudomonas*, *Psychrobacter*, and *Sphingomonas*. We determined, in three stages of visual analytics views, the distribution of denitrification potential patterns for 2603 genomes from the nine bacteria genera (Figure 7). Our categorization method assigned a complete denitrification pattern to 1331 genomes from four genera (*Arcobacter*, *Bradyrhizobium*, *Pseudoalteromonas*, and *Pseudomonas*). Furthermore, the following six genomes were assigned to the Mollusca (shellfish) ecosystem category: *Arcobacter ellisii* LMG 26155, *Arcobacter ellisii* CECT 7837, *Arcobacter venerupis* CECT7836, *Arcobacter* sp. LA11, *Pseudomonas alcaligenes* OT 69, and *Psychrobacter* sp. C 20.9. Only the *Arcobacter* sp. LA11 genome had the complete denitrification trait (Figure 7) with a denitrifying enzymes pattern of “000110111001” (presence in genome of *napA*, *napB*, *nirS*, *norB*, *norC*, and *nosZ*). *Arcobacter* sp. LA11, which was isolated from the gut of the abalone *Haliotis discus*, has the complete repertoire genes for nitrogen fixation and denitrification [56]. *Pseudoalteromonas denitrificans* DSM 6059, a denitrifying marine bacterium [57], has the same denitrifying enzymes pattern as *Arcobacter* sp. LA11 (Figure 7c).

**Figure 8:**
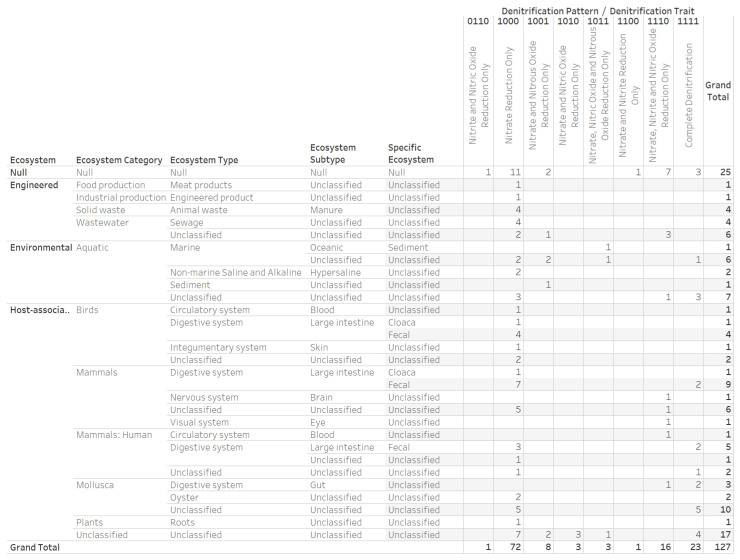
Ecosystem classifications and denitrification potential patterns of 127 *Arcobacteraceae* genomes. The association of *Arcobacteraceae* with multi-ecosystem habitats including human, animal, plants, and the environment presents a bacteria family for research on synthetic denitrifying communities.

The findings on *Arcobacter* genomes with complete denitrification traits (Figure 7a) as well as the recommendation for research on *Arcobacter* strains and their hosts [56] led us to construct a denitrification potential dataset for 127 genomes taxonomically classified to the bacteria family *Arcobacteraceae*. The ecosystem classification and counts of genomes according to denitrification potential revealed *Arcobacteraceae* strains inhabit engineered, environmental, and host-associated ecosystems (Figure 8). *Arcobacteraceae* family members are associated with diverse ecosystem categories (including human, animals, plants, wastewater, marine and non-marine aquatic environments, food production, and industrial production).

The digital categorization assigned the 127 *Arcobacteraceae* genomes to eight of the sixteen denitrification potential traits. The eight categories and associated number of genomes were as follows: (1) nitrite and nitric oxide reduction only (1 genome); (2) nitrate reduction only (74 genomes); (3) nitrate and nitrous oxide reduction only (8 genomes); (4) nitrate and nitric oxide reduction only (4 genomes); (5) nitrate, nitric oxide, and nitrous oxide reduction only (3 genomes); (6) nitrate and nitrite reduction only (1 genome); (7) nitrate, nitrite, and nitric oxide reduction only (19 genomes); and (8) complete denitrification (23 genomes). Among the *Arcobacteraceae* genomes investigated, only the *Aliarcobacter cryaerophilus* AZT-1 genome (denitrifying enzymes pattern “000010111000”) did not encode the periplasmic nitrate reductase complex NapAB, as only the gene for NapB was present. The IMG/M annotated the ecosystem category of Mollusca to 15 *Arcobacteraceae* genomes in three denitrification traits categories of complete denitrification (7 genomes); nitrate reduction only (7 genomes); and nitrate, nitrite, and nitric oxide reduction only (1 genome) (Table 3).

### 3.4. Nitrogen Assimilation, Taxonomic, and Ecosystems Annotations for Genomes with a Complete Denitrification Pattern

We uploaded to the IMG/M system the list of 3280 identifiers (“taxon_oid”) for the genomes with a complete denitrification pattern. We then used the IMG/M Find Function tool to identify genomes that have genes for four nitrogen assimilation pathways. The pathways investigated were nitrogen fixation, assimilatory nitrate reduction, assimilatory nitrite reduction, and ammonia assimilation to glutamine [5,10]. There were 369 bacteria genomes that encoded *nifH*, a biomarker used for identifying nitrogen-fixing bacteria and archaea [58,59] (Table 4). In addition, 3164 of the 3280 bacterial genomes (96.5%) had the glutamine synthetase (*glnA*) gene (KEGG Entry: K01915) for ammonia assimilation to glutamine. The examples of bacterial strains provided in Table 4 are from an ecosystem perspective, including an example relevant to Mollusca health. *Aliiroseovarius crassostreae* DSM 16950, a causative bacterium of *Roseovarius* oyster disease in Eastern oysters (*Crassostrea virginica*), is an example of 664 complete denitrifying bacterial strains encoding the *glnA* and without evidence for genes of the four other nitrogen assimilatory pathways investigated. We also searched the IMG/M database for genomes that we assigned the complete denitrification pattern and annotated with the KEGG identifier of K01601 (ribulose-bisphosphate carboxylase large chain [EC:4.1.1.39]) for carbon fixation. The genomes of three strains (CECT 5094, CECT 5095, and CECT 5096) of *Roseibium album* isolated from oysters were among the 695 genomes annotated with the gene for carbon fixation.

**Figure 9:**
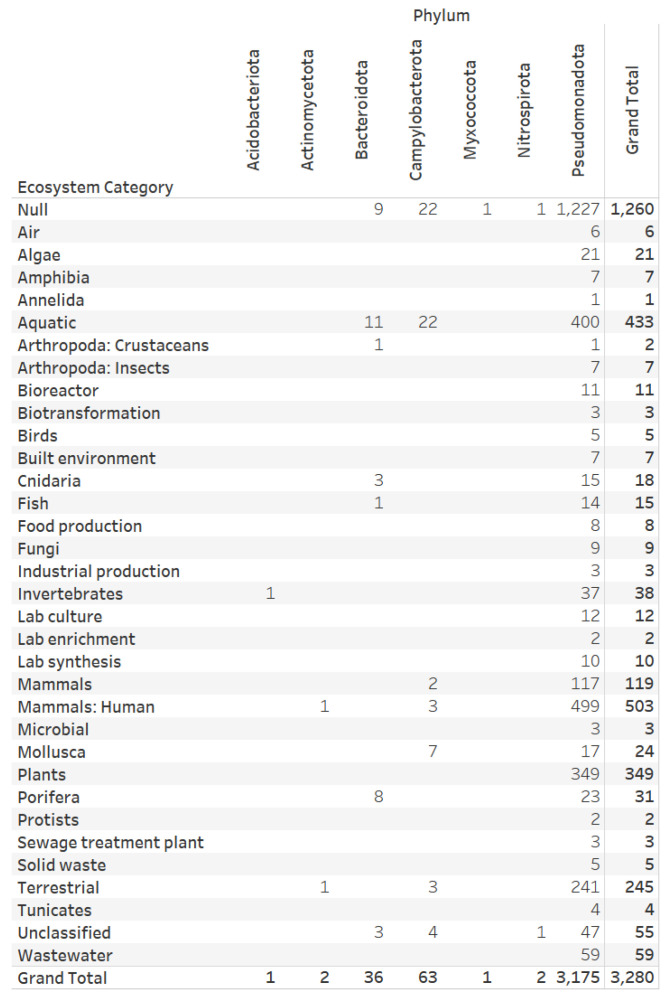
Ecosystem categories assigned to 3280 bacterial genomes with complete denitrification potential. The phyla Campylobacterota and Pseudomonadota have genera associated with Mollusca (shellfish).

Several authors have recognized oyster-mediated denitrification as a long-term removal of reactive nitrogen (e.g., nitrate) from coastal ecosystems [60,61]. We are especially interested in genomes from phyla with the complete denitrification pattern and annotated with the Mollusca ecosystem category. Therefore, we designed a visual analytics view that integrates taxonomic and ecosystem category annotations for the 3280 bacteria genomes categorized to the complete denitrification potential pattern (“1111”). The genomes with complete denitrification patterns were from 260 bacteria genera, of which 256 genera are validly published. We observed seven bacteria phyla: Acidobacteriota (1 genome), Actinomycetota (2 genomes), Bacteroidota (36 genomes), Campylobacterota (63 genomes), Myxococcota (1 genome), Nitrospirota (2 genomes), and Pseudomonadota (3175 genomes) (Figure 9). The phyla Campylobacterota (6 classified taxonomic families) and Pseudomonadota (71 classified taxonomic families) have genera associated with Mollusca (shellfish). The Campylobacterota families with genomes with complete denitrification potential are *Arcobacteraceae* (23 genomes), *Helicobacteraceae* (1 genome), *Hydrogenimonadaceae* (1 genome), *Nitratiruptoraceae* (10 genomes), *Sulfurimonadaceae* (20 genomes), and *Sulfurovaceae* (5 genomes).

**Figure 10:**
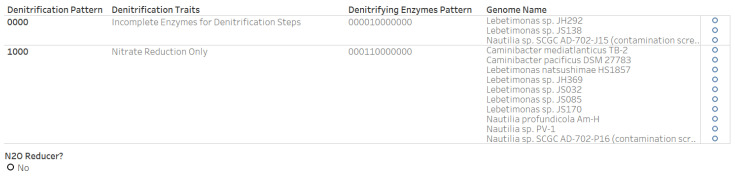
Evidence from binary numbering patterns indicating that three Campylobacterota genera (*Caminibacter*, *Lebetimonas*, and *Nautilia*) do not encode the gene for nitrous oxide reductase. The last digit of the “Denitrification Pattern” and “Denitrifying Enzymes Pattern” is “0”.

Since *Arcobacteraceae* is a member of the Campylobacterota and since denitrifying *Arcobacteraceae* strains have been isolated from oysters (Figure 9), we conducted a literature search on the other Campylobacterota families with genomes categorized as having complete denitrification potential. Our search retrieved a publication on nitrous oxide reducing Campylobacterota isolated from deep-sea hydrothermal environments [62]. The Campylobacterota genera listed in the publication as having strains with potential nitrous oxide reducers are *Nitratifractor*, *Nitratiruptor*, *Sulfurimonas*, and *Sulfurovum*. The availability of a comparative set of Campylobacterota genera (*Lebtimonas*, *Nautilia*, and *Caminibacter*) whose strains do not reduce nitrous oxide allowed us to verify the accuracy of the binary data synthesis of the denitrification dataset. In the denitrification dataset, the potential nitrous oxide reducers had “1” while non-nitrous oxide reducers had “0” in the last digit of the twelve-digit denitrifying enzymes pattern and four-digit denitrification pattern. The last digit for the two patterns was “0” for the *Lebtimonas*, *Nautilia*, and *Caminibacter* genomes (Figure 10).

**Figure 11:**
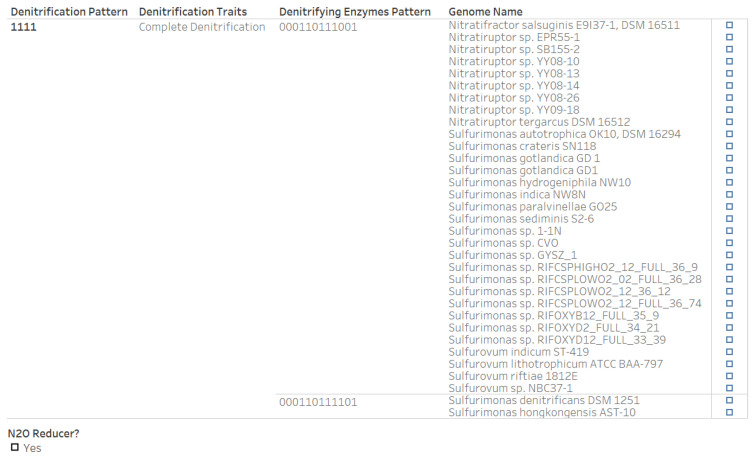
Genomes of the genera in phylum Campylobacterota (*Nitratifractor*, *Nitratiruptor*, *Sulfurimonas*, and *Sulfurovum*) that have the complete denitrification pattern (“1111”) in the microbial denitrification potential dataset. The nitrous oxide reductase activity of strains from the taxonomic class campylobacteria associated with deep-sea hydrothermal vents was reported by Fukushi et al. [62].

Among the Campylobacterota genera that are potential nitrous oxide reducers, *Sulfurovum* and *Sulfurimonas* have genomes that encode and those that do not encode nitrous oxide reductase. Additionally, 33 genomes with the complete denitrification pattern include all one of the *Nitratifractor* and eight of the *Nitratiruptor* strains as well as twenty of the *Sulfurimonas* and four of the *Sulfurovum* strains (Figure 11).

### 3.5. Searches for Scholarly Articles with Gene Symbols of Enzymes for Denitrification

**Figure 12:**
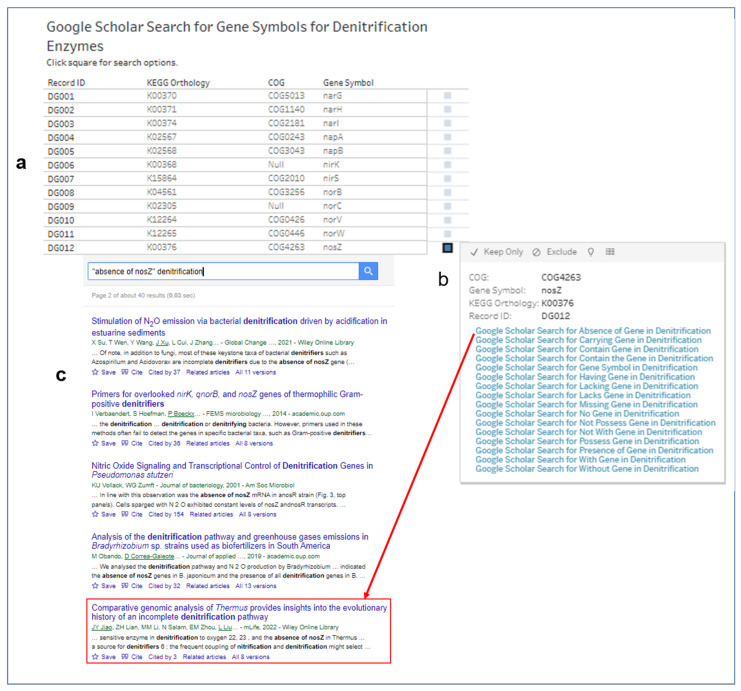
Visual interfaces for selecting and exploring searches for scholarly articles with gene symbols of enzymes for denitrification. (**a**) The list of functional annotation identifiers and gene symbol for enzymes in the canonical denitrification pathway. Selecting the square for each gene symbol displays the Google Scholar search options. (**b**) The list of search text for Google Scholar to retrieve up-to-date journal articles and other scholarly literature. (**c**) An example of part of the retrieved results for the search text “(‘absence of nosZ’ denitrification)”. The selected journal article provides insights into the evolutionary history of the incomplete denitrification pathway of the bacteria genus, *Thermus*.

We designed a visual analytics worksheet that lists the gene symbols and other identifiers for the 12 denitrifying enzymes (Figure 12a). Additionally, the design included uniform resource locator (URL) actions for 16 Google Scholar searches, with the prefix text “denitrification” and the gene symbol (e.g., “*narG*“ and “*nosZ*”) of the denitrifying enzymes being part of the design (Figure 12b). When a researcher selects the Google Scholar URL action, the results will be up to date, with options to retrieve related articles and articles citing the retrieved article. The URL action might also retrieve the context of the search text within the scholarly article. The search texts that include the gene symbol prefixed with negation words (such as “absence”, “lack”, “lacking”, “missing”, “no”, “not possess”, “not with”, and “without”) can retrieve scholarly articles on incomplete denitrification. A Google Scholar search with search text “(‘absence of nosZ’ denitrification)” retrieved 40 results as of 23 March 2024, including an article on incomplete denitrification trait for 23 *Thermus* strains associated with terrestrial geothermal environments [63] (Figure 12c).

**Figure A2:**
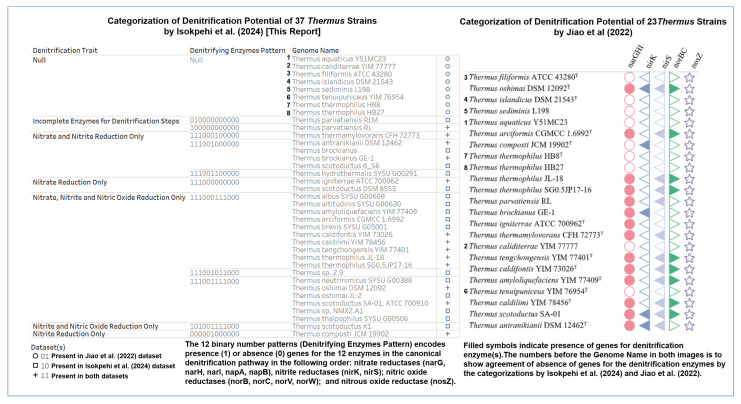
Comparison of the categorizations of *Thermus* strains in this report (Isokpehi et al., 2024) and those of Jiao et al. (2022) [63]. Among the 23 *Thermus* strains, eight strains are not included in our microbial denitrification potential dataset because they do not have at least one of the genes for the 12 enzymes for denitrification. In the categorization by Jiao et al. [63], the filled symbols indicate presence of genes for denitrification enzymes. The numbers before the Genome Name in both images is to show agreement of absence of genes for the denitrification enzymes by the categorizations by Isokpehi et al. (2024) and Jiao et al. [63]. In addition, the list of *Thermus* strains include Type strains (with superscript “T”). The open access image by Jiao et al. is available at https://doi.org/10.1002/mlf2.12009 (accessed on 23 March 2024).

We used this list of strains from the scholarly article by Jiao et al. [63] to determine the overlap with the 29 genomes of *Thermus* strains in the microbial denitrification potential dataset. According to the article, the 23 genomes of *Thermus* do not encode the gene for nitrous oxide reductase (nosZ). An explanation for the absence is that nosZ is sensitive to oxygen. The absence of nosZ gene is consistent with the denitrification patterns and denitrification trait assigned by our study (Appendix B, Figure A2). Furthermore, the *Thermus* genomes absent in our dataset were reported by Jaio et al. [63] as lacking the genes for the denitrification pathway. Thus, the data-investigation interfaces supported knowledge discovery on nosZ biochemical characteristics and evolutionary history through a combination of (1) scholarly searchers, (2) the presence or absence of genes for denitrifying enzymes in genomes, and (3) patterns of denitrification traits.

The article by Jiao et al. [63] also notes the presence of nosZ in the genome of the related bacteria, *Deinococcus ficus* CC-FR2-10. There are six *Deinococus* genomes in our microbial denitrification potential dataset, of which *Deinococcus ficus* CC-FR2-10 encodes the genes for nitrite reductase (nirK) and nosZ. We interpreted the presence of only nirK and nosZ genes as the denitrification trait of “Nitrite and Nitrous Oxide Reduction Only”. The other three *Deinococus* genomes assigned to the same denitrification trait in our dataset are *Deinococcus enclensis* DSM 25127, *Deinococcus ficus* DSM 19119, and *Deinococcus ficus* KS 0460. The remaining two *Deinococcus* genomes (*Deinococcus* sp. NW-56 and *Deinococcus yavapaiensis* DSM 18048) have denitrification trait “Nitrite Reduction Only”.

### 3.6. Denitrification Patterns of Archaeal Genomes

**Figure A3:**
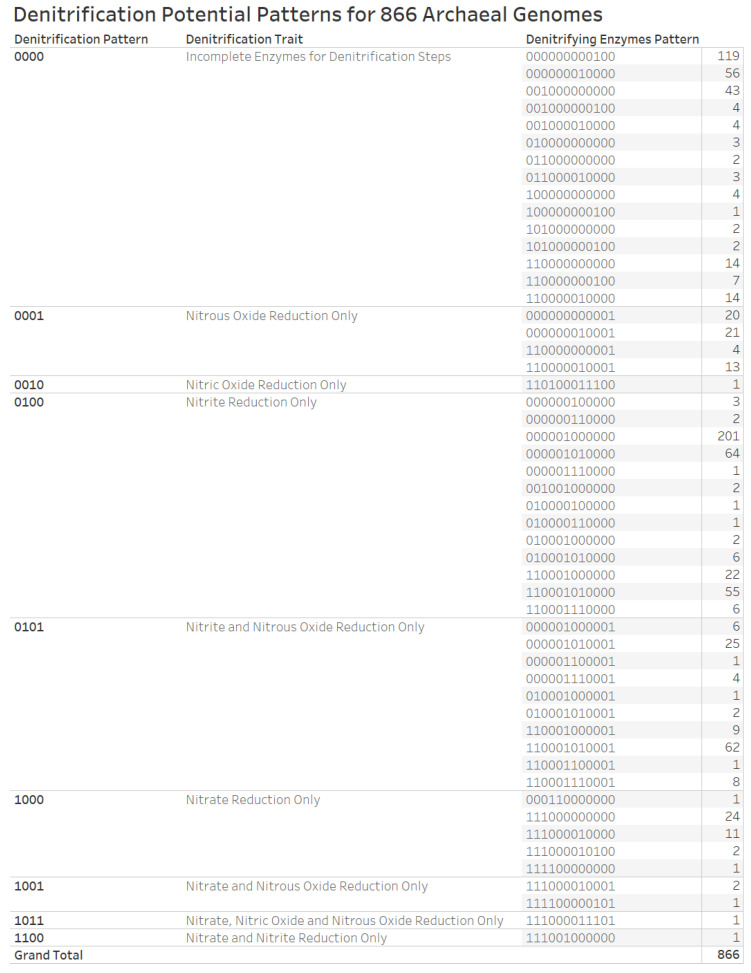
The categories of denitrification-potential patterns for 866 archaeal genomes. The genomes encode at least 1 of the 12 enzymes in the canonical four-step denitrification pathway according to the MicroTrait rules (Figure A1). The 12-digit binary numbers represent the presence (“1”) or absence (“0”) of the following enzymes: narG, narH, narI, napA, napB, nirK, nirS, norB, norC, norV, norW, and nosZ. A pattern of interest can be used to search the interactive version of Figure 5 available at https://public.tableau.com/app/profile/qeubic/viz/microbial_denitrifiers/abstract/ (accessed on 23 March 2024).

The 866 archaeal genomes were assigned to 9 of possible 16 denitrification patterns. These nine denitrification patterns were deduced from 52 twelve-digit binary number codes (Table 5 and Appendix C Figure A3). None of the archaeal genomes had a complete denitrification pattern. The potential for nitrate reduction (represented by “1” in the first three digits of the twelve-digit binary number) was assigned to 43 genomes including *Ferroglobus placidus* AE-DII12DO, DSM 10642, the only member of a denitrification pattern that has the denitrification potential for “Nitrate, Nitric Oxide and Nitrous Oxide Reduction Only”. The other one-archaea member denitrification potential categories were (1) “Nitric Oxide Reduction Only” (*Candidatus* Hydrothermarchaeota archaeon JdFR-18), and (2) “Nitrate and Nitrite Reduction Only” (*Candidatus* Heimdallarchaeota archaeon LC_3). Among the archaeal genomes investigated, 585 genomes encoded the nitrate reduction trait (represented by the sixth digit and seventh digit in the twelve-digit binary number). The *Ferroglobus placidus* genome did not encode *nirK* or *nirS* for nitrite reduction to produce nitric oxide, consistent with findings from a publication on the genome sequence of the archaea [64]. In addition, the genome of *Ferroglobus placidus* had gene annotations for carbon fixation (K01601) and glutamine synthetase (K01915). Table 5 includes references to research on the denitrification potential of the example archaea genome. The microbial denitrification dataset contains 21 archaeal genomes (7 genera and 18 unique strains) that encode both nirK and nirS genes for nitrite reduction. The seven Halobacteriota genera are *Halobiforma*, *Halorubrum*, *Halosolutus*, *Haloterrigena*, *Natrinema*, *Natronomonas*, and *Salinilacihabitans* (Table 6).

## 4. Discussion

In this study, we investigated the denitrification potential in the context of taxonomic and ecosystem features for 62,624 microbial genomes (866 archaea and 61,758 bacteria). The dataset constructed includes 181 archaeal and 8009 bacterial genomes with the nitrous oxide reductase gene (nosZ) (Table 2). This fundamental scientific knowledge of archaea and bacteria includes trait knowledge (e.g., complete denitrification), which is needed for machine learning models that scale knowledge at microsites for decision-making at a global scale [74]. Incomplete microbial denitrification that results in the production and emission of harmful nitrous oxide gas is detrimental to the health of humans, animals, plants, and the environment [75]. Nitrous oxide reductase catalyzes the last step of denitrification, which transforms the ozone-layer-depleting nitrous oxide to dinitrogen gas [75,76,77]. Our research builds on the microTrait categories [10] and the 2019 publication by Albright et al. [5] that reported the presence of annotations for 11 nitrogen cycling pathways in 6384 bacterial and 252 archaeal finished genomes in the IMG/M database. The collection of the IMG/M genomes investigated in our study includes three categories of genome sequencing status: draft, finished, and permanent draft. The constructed microbial denitrification potential dataset also includes taxonomic and ecosystem annotations of the genomes. Some strains (e.g., *Brucella melitensis* bv. 1 16 M with complete denitrification trait) have more than one genome sequence available in IMG/M, allowing for the produced dataset to include biological and technical replicates. This unique denitrification potential dataset is useful for planning and conducting microbiological research on denitrification. The methods implemented in the data investigation can be adapted for traits defined by ecological functions of resource acquisition, resource use, and stress tolerance [10], for example, the microbial genes involved in the resource acquisition function of nitrogen fixation, where microorganisms convert atmospheric nitrogen gas to biologically available ammonia [59]. The categorization for nitrogen fixation potential can be based on the presence or absence in genome of a set of six genes (nifH, nifD, nifK, nifE, nifN, and nifB) coding for structural and biosynthetic components, namely NifHDK and NifENB [58].

The microbial denitrification dataset allows researchers to retrieve subsets of bacteria or archaea strains with 1 or more of 36 variables (Table 1). A query of the dataset with keyword “denitrificans” in the “Genome Name” field combined with environment ecosystem and complete denitrification pattern (“1111”) retrieved 20 genomes (Figure 5). The possibility for human interaction with the dataset can facilitate the production of evidence by comparison of the digits in the 4-digit binary “Denitrification Pattern” and 12-digit binary “Denitrifying Enzymes Pattern”. Digit 6 and Digit 7 in the 12-digit pattern are, respectively, for the presence or absence of the gene for copper-type nitrite reductase (*nirK*) and the gene for cytochrome cd1-type nitrite reductase (*nirS).* In the case of aquatic ecosystems, aquatic bacteria inhabit a variety of microhabitats such as diffusion-controlled water phases, colloidal phases, particles, and within the living biosphere (oyster tissue, zooplankton, algae, fish, etc.), which are impacted by and also influence abiotic factors within the water and/or tissues they inhabit [78]. The gaseous nitric oxide is an intermediate product of the rate-limiting step of denitrification [79]. The possibility that nitric oxide can be an extracellular signaling molecule between aerobic bacteria (e.g., *Phaeobacter inhibens*) and algae (e.g., *Gephyrocapsa huxleyi*) [80] presents the use for our data resources to investigate the denitrification potential of aerobic marine bacteria. Bacterial *nirK* is expressed in oxygenated marine waters that have detectable nitrite levels and photosynthesizing microorganisms [80]. The microbial denitrification potential dataset contains 49 *Phaeobacter* from 47 strains, with 43 genomes having evidence of *nirK* for the reduction of nitrite to nitric oxide.

A study of a collection of 249 archaeal genomes (170 Euryarchaeota, 65 Crenarchaeota, and 14 Thaumarchaeota) reported only partial denitrification pathways (nitrite reduced to nitric oxide, nitric oxide to nitrous oxide, and nitrous oxide to nitrogen gas) [5]. In our study of 866 archaeal genomes (Table 5), we found genomic evidence for three denitrification steps for the metabolic versatile *Ferroglobus placidus* AEDII12DO, a hyperthermophilic, strictly anaerobic chemolithoautotroph iron-oxidizer that belongs to the *Archaeoglobaceae* family in the phylum Euryarchaeota. The genome sequence of strain AEDII12DO does not have annotations for the nitrite reductases (nirK or nirS) that produce nitric oxide in the second stage of denitrification [64,81]. In cells of aerobic ammonia-oxidizing archaea (AOA), the highly reactive nitric oxide is needed for sustaining aerobic ammonia oxidation activity [82]. We identified 21 archaea genomes (7 genera and 18 strains) of the phylum Halobacteriota that encode both nitrite reductases (Table 6). In the case of bacteria genomes with both genes, our dataset contains 257 bacterial genomes from at least 57 genera including (1) *Methyloprofundus* associated with the gills of the mussel, *Bathymodiolus platifrons* [83] and (2) the oligotrophic nitrogen-fixing *Bradyrhizobium oligotrophicum* S58 [84]. The presence of two types of nitrate reductases could confer archaea and bacteria with the potential to produce nitric oxide in different saline environments of (1) non-saline and low salinity (rivers and fresh water lagoons), (2) slight and moderate salinity (oceans, estuaries and mangroves), and (3) hypersalinity (salt marshes, hypersaline lakes, and salty ponds) [85]. One of the nitrite reductases may also function beyond denitrification, such as in the colonization of rice roots by *Bradyrhizobium oligotrophicum* S58 through maintaining swimming motility under fluctuating oxygen conditions in the presence of nitrate [84]. Thus, the type of nitrite reductase encoded in a microbial genome could be predictive of the microbe’s ecological functioning [82,85,86].

Growing anthropogenic disturbances, including climate change, invasive species, and micro/nanoplastics, are likely influencing microbial communities and impacting microbial processes [87,88]. This dataset will assist researchers in identifying changes in denitrification potentials that may occur with changes in microbial diversity due to disturbance. In addition to the availability of genomic sequences of single microbial isolates, metagenomics sequencing technologies produce data on the microbiome (the collective set of gene sequences from multiple genomes) in a specific habitat and timeframe [89]. Microbiome/metagenomic analyses of ecosystems such as engineered (e.g., wastewater), environmental (e.g., soil and seawater), and host-associated (e.g., oyster) types have revealed constituent microorganisms as well as the enzyme genes for denitrification [37,39,40,41,42,90]. We suggest that the data-investigation products (Supplemental Materials) can be useful for producing evidence on the denitrification patterns of identified taxa from microbiome analysis. For example, a microbiome analysis of the Eastern oyster as a function of ploidy and seasons identified metagenomics associated genome *Psychrobacter maritimus* as having genes for denitrifying enzyme genes *narH*, *narI*, *nirK*, and *norB* [42]. The patterns for the *Psychrobacter maritimus* Pi2-25 denitrification dataset have the denitrification pattern “1100” (nitrate and nitrite reduction only) and denitrifying pattern “111001010010” (presence of *narG*, *narH*, *narI*, *nirK*, *norB*, and *norW*).

We designed and implemented interactive visualizations in visual analytics software for two main purposes related to microbial denitrification. The first purpose is to provide evidence for microbial denitrification potential by comparing patterns of presence or absence in microbial genomes of denitrifying enzymes for ecologically relevant denitrification trait standards (Figure 5, Figure 6 and Figure 7). The second purpose is to facilitate personalized and collaborative learning and knowledge exchange on microbial denitrification by connecting to bioinformatics and scholarly resources. The inclusion of hyperlinks in the visual analytics design allows for the 62,624 genome names in the denitrification dataset to be searched with search engines and literature databases that are up to date (Figure 5 and Figure 6). A major contribution of our data investigations is a denitrification potential categorized dataset of microbial genomes that allows for decision-making on the choice of microbial strains for sustainable microbial denitrification applications. For example, a recent report experimentally combined two denitrifying bacteria strains, *Paracoccus denitrificans* PD1222 and *Ochrobactrum* sp. TCC-2, to mitigate nitrous oxide emission and detoxify triclocarban, a widespread broad-spectrum antimicrobial [91]. Our microbial denitrification dataset contains 96 strains of *Paracoccus* and 77 strains of *Ochrobactrum* (including those previously classified as the *Bacillus* genus). The counts of strains with complete denitrification patterns were 43 and 34 for *Paracoccus* and *Ochrobactrum*, respectively.

The constructed dataset and accompanying interactive data-investigation resources can help to advance research into the molecular, biochemical, physiological, and microbial aspects of denitrification, among others. The total 12-digit “Denitrifying Enzymes Patterns” observed in the 62,624 genomes were 484 out of possible 4096 twelve-digit patterns. We have provided the microbial denitrification dataset in a variety of data formats (comma separated file, spreadsheet, and Tableau views) for further data investigations, research, applications, and education purposes. Following the guidelines for constructing ecological trait datasets [12], the microbial denitrification dataset contains identifiers for connecting to microbial web portals and scholarly resources. The microbial denitrification potential dataset, spreadsheet files, and interactive visual analytics resources are available as online or off-line tools to articulate the value of data. Researchers can incorporate these denitrification-potential data resources into research on the biochemical, molecular, and physiological aspects of denitrification, among others. For example, when a research team is describing a new bacteria or archaea isolate or genome sequence for publication, researchers could compare the denitrification-potential patterns of the isolate with members of the same genus in our microbial denitrification dataset.

Several *Arcobacteraceae* strains are associated with the Mollusca ecosystem category and include strains with complete denitrification potential (Figure 7). Although there have been discussions on the nomenclature changes and new genera described, there is a consensus that the *Arcobacteraceae* family is justified [92,93]. *Arcobacteraceae* strains have been isolated from diverse habitats including terrestrial, aquatic, animal, food, and human [92,93,94,95,96]. The presence of antimicrobial resistance genes has been documented in strains of *Arcobacteraceae* [94]. Antimicrobials such as triclocarban that occur with anthropogenic reactive nitrogen sources in the environment can affect the efficiency of denitrification [91]. There is a need to investigate denitrifying *Arcobacteraceae* for effects of antimicrobials on denitrification rates. In addition, using studies of synthetic denitrifying communities of *Shewanella* as a guide [7], we suggest investigations into synthetic denitrifying communities of *Arcobacteraceae* for optimized and stable denitrification in ecosystems.

There are limitations of this data-investigation project. The datasets used in the project are from different sources, and data providers might complete updates as new data become available. For example, the bacterial taxonomic classification may be updated or be inconsistent between methods of annotation. To mitigate this limitation, we have included multiple taxonomic sources as well as web links to Integrated Microbial Genomes and Microbiomes (IMG/M). We based the digital categorization of denitrification potential on 12 enzymes in the canonical denitrification pathway. In some cases, we verified the accuracy of the patterns using published studies [62,63] that tested for the presence of denitrification enzymes. However, other factors can affect the functional performance of the denitrification trait such as environmental and genetic factors [74,97]. Our principal data source for the datasets is IMG/M from genomes of varying levels of genome sequence completion (finished, draft, and permanent draft). Therefore, we have included a filter on the genome sequencing status in some views to help researchers decide on the data to use.

## 5. Conclusions

Denitrification is a major component of the nitrogen cycle for the reduction of harmful nitrous oxide gas to harmless dinitrogen gas. We articulated the denitrification potential in context of taxonomic classification and ecosystem features for 62,624 microbial genomes (866 archaea and 61,758 bacteria). We recommend denitrification traits of *Arcobacteraceae* for further research because of (1) the bacteria family’s global distribution; (2) associations with humans, animals, plants, and the environment; (3) presence of antimicrobial resistance genes; (4) assignment of 127 genomes to eight denitrification traits, and (5) the interaction of some *Arcobacteraceae* strains with shellfish filter feeders. Finally, the microbial denitrification data resources produced in our research can also be useful for identifying microbial strains for synthetic denitrifying communities.

## Figures and Tables

**Table 1:** Data columns in the microbial denitrification potential dataset including genome annotations and denitrification annotations.

Dataset Column Category ^1^	Dataset Columns
Genome	Domain, Gene Count, Genome ID, Genome Name, Genome Size, Genus, GOLD Sequencing Project ID, Sequencing Center, Sequencing Status, Status
Ecosystem	Ecosystem, Ecosystem Category, Ecosystem Type, Ecosystem Subtype, Specific Ecosystem
Lineage	GTDB-Tk Domain, GTDB-Tk Family, GTDB-Tk Genus, GTDB-Tk Order, GTDB-Tk Phylum, GTDB-Tk Species
Denitrifying Enzymes	E01_narG, E02_narH, E03_narI, E04_napA, E05_napB, E06_nirK, E07_nirS, E08_norB, E09_norC, E10_norV, E11_norW, E12_nosZ
Denitrification Potential	Denitrification Pattern, Denitrification Traits, Denitrifying enzymes pattern

^1^ Genome, Ecosystem and Lineage categories were retrieved from the Integrated Microbial Genomes and Microbiomes (IMG/M) system. Denitrifying enzymes and denitrification potential were derived/calculated in visual analytics software based on the datasets of genomes with KEGG Orthology annotation in the IMG/M system.

**Table 2:** Functional annotation identifiers, gene nomenclature of enzymes, and count of genomes in associated with canonical denitrification pathway.

KEGG Orthology (KO) Identifier ^1^	KEGG Gene Name	Gene Symbol ^2^	Genome Count ^3^		
			Archaea	Bacteria	Total
K00370	nitrate reductase/nitrite oxidoreductase, alpha subunit	*narG*	268	39,961	40,229
K00371	nitrate reductase/nitrite oxidoreductase, beta subunit	*narH*	280	39,985	40,265
K00374	nitrate reductase gamma subunit	*narI*	105	41,172	41,277
K02567	nitrate reductase (cytochrome)	*napA*	4	21,911	21,915
K02568	nitrate reductase (cytochrome), electron transfer subunit	*napB*	1	21,754	21,755
K00368	nitrite reductase (NO-forming)	*nirK*	479	10,873	11,352
K15864	nitrite reductase (NO-forming)/hydroxylamine reductase	*nirS*	28	3205	3233
K04561	nitric oxide reductase subunit B	*norB*	364	15,680	16,044
K02305	nitric oxide reductase subunit C	*norC*	2	6163	6165
K12264	anaerobic nitric oxide reductase flavorubredoxin	*norV*	138	17,164	17,302
K12265	nitric oxide reductase FlRd-NAD(+) reductase	*norW*		14,224	14,224
K00376	nitrous-oxide reductase	*nosZ*	181	8009	8190

^1^ The Kyoto Encyclopedia of Genes and Genomes (KEGG) database was the source of the identifiers. ^2^ The list of enzyme genes was obtained from the denitrification trait rules that are based on the presence or absence of a protein family in a microbial genome [10]. ^3^ Data were retrieved from the Integrated Microbial Genomes and Microbiomes (IMG/M) system in November 2023.

**Table 3:** Denitrification traits of selected *Arcobacteraceae* genomes isolated from Mollusca hosts.

Denitrification Trait	Genome Name (Mollusca Host) ^1^
Complete Denitrification	*Arcobacter* sp. LA11 (abalone) *Halarcobacter mediterraneus* F156-34 (mussel) *Malaciobacter mytili* CECT 7386 (mussel) *Malaciobacter mytili* F2075 (mussel) *Poseidonibacter parvus* LPB0137 (squid) *Poseidonibacter* sp. SJOD-M-5 (oyster) *Poseidonibacter* sp. SJOD-M-33 (oyster)
Nitrate Reduction Only	*Arcobacter ellisii* LMG 26155 (mussel) *Arcobacter venerupis* CECT7836 (clam) *Malaciobacter canalis* F138-33 (oyster) *Malaciobacter canalis* LMG 29148 (oyster) *Malaciobacter molluscorum* CECT 7696 (mussel) *Malaciobacter molluscorum* F98-3 (mussel)
Nitrite and Nitric Oxide Reduction Only	*Poseidonibacter ostreae* JOD-M-6 (oyster)

^1^ The details for each genome are available from the Integrated Microbial Genomes and Microbiomes (IMG/M) system website.

**Table 4:** Distribution of nitrogen assimilation pathways for 3280 bacterial genomes assigned with a complete denitrification pattern.

Nitrogen Assimilation Pathway	KEGG Entry and Name ^1^	Gene Symbol	Genome Count	Example Genome
Nitrogen Fixation	K02588 nitrogenase iron protein	*nifH*	369	*Arcobacter acticola* KCTC 52212
Assimilatory Nitrate Reduction	K00372 assimilatory nitrate reductase catalytic subunit [EC:1.7.99.-]	*nasA*	2294	*Shewanella denitrificans* OS217
Assimilatory Nitrate Reduction	K00360 assimilatory nitrate reductase electron transfer subunit [EC:1.7.99.-]	*nasB*	2	*Halomonas icarae* D1-1
Assimilatory Nitrate Reduction	K00367 ferredoxin-nitrate reductase [EC:1.7.7.2]	*narB*	47	*Sulfuricella denitrificans* skB26
Assimilatory Nitrite Reduction	K00366 ferredoxin-nitrite reductase [EC:1.7.7.1]	*nirA*	170	*Arcobacter peruensis* PSE-93
Ammonia Assimilation to Glutamine	K01915 glutamine synthetase [EC:6.3.1.2]	*glnA*	3164	*Aliiroseovarius crassostreae* DSM 16950

^1^ The Kyoto Encyclopedia of Genes and Genomes (KEGG) database was the source of the identifiers.

**Table 5:** Denitrification potential patterns observed for 866 archaeal genomes.

Denitrification Pattern ^1^	Denitrification Trait (Count of Denitrifying Enzymes Pattern) ^2^	Genome Count	Example Genome	Denitrifying Enzymes Pattern of the Example Genome ^3^	Reference for the Denitrification Pattern
0000	Incomplete Enzymes for Denitrification Steps (15)	278	*Aeropyrum pernix* K1	110000000000	[65,66]
0001	Nitrous Oxide Reduction Only (4)	58	*Haloarcula japonica* DSM 6131	110000010001	[67]
0010	Nitric Oxide Reduction Only (1)	1	*Candidatus* Hydrothermarchaeota archaeon JdFR-18	110100011100	[68]
0100	Nitrite Reduction Only (13)	366	*Haloferax volcanii* DS2	110001010000	[69]
0101	Nitrite and Nitrous Oxide Reduction Only (10)	119	*Haloferax mediterranei* R-4	110001010001	[70]
1000	Nitrate Reduction Only (5)	39	*Pyrobaculum aerophilum* IM2	111000010000	[71]
1001	Nitrate and Nitrous Oxide Reduction Only (2)	3	*Pyrobaculum calidifontis* JCM 11548	111000010001	[71]
1011	Nitrate, Nitric Oxide and Nitrous Oxide Reduction Only (1)	1	*Ferroglobus placidus* AEDII12DO, DSM 10642	111000011101	[64,72]
1100	Nitrate and Nitrite Reduction Only (1)	1	*Candidatus* Heimdallarchaeota archaeon LC_3	111001000000	[73]

^1^ The 4-digit binary number encodes the denitrification traits (second column in the Table) according to the MicroTrait rules for denitrification [10]. ^2^ Details of the 52 “Denitrifying enzymes pattern” are provided in Appendix C Figure A3. ^3^ The 12-digit binary number encodes the presence (“1”) or absence (“0”) of denitrifying enzymes in the following order narG, narH, narI, napA, napB, nirK, nirS, norB, norC, norV, norW, and nosZ.

**Table 6:** Archaeal genomes with genes for copper-type nitrite reductase (nirK) and cytochrome cd1-type nitrite reductase (nirS).

Genome Name	Genome ID ^1^	Denitrifying Enzymes Pattern ^2^	Ecosystem Category
*Halobiforma haloterrestris* DSM 13078	2693429869	110001110001	Terrestrial
*Halobiforma lacisalsi* AJ5, JCM 12983	2529293100	110001110001	Aquatic
*Halobiforma lacisalsi* AJ5, JCM 12983	2806310686	110001110001	Aquatic
*Halobiforma nitratireducens* JCM 10879	2554235466	110001100001	Aquatic
*Halorubrum amylolyticum* ZC67	2881047951	110001110001	Terrestrial
*Halorubrum salipaludis* WN019	2995789858	000001110001	Terrestrial
*Halosolutus halophilus* LT55	8055007790	000001110001	Terrestrial
*Haloterrigena longa* ABH32	8065811630	000001110001	Aquatic
*Haloterrigena* sp. LL2A	2639762614	000001110001	Aquatic
*Natrinema altunense* AJ2	2585427993	110001110001	Aquatic
*Natrinema altunense* JCM 12890	2554235488	110001110001	Aquatic
*Natrinema amylolyticum* LT61	8056733939	110001110001	Terrestrial
*Natrinema pallidum* BOL6-1	8058325716	110001110000	Terrestrial
*Natrinema pellirubrum* 157	2509601048	110001110000	Fish
*Natrinema pellirubrum* 157	2537562080	110001110000	Fish
*Natrinema* sp. J7-2	2517093029	000001110000	Terrestrial
*Natrinema thermotolerans* A29	2582580504	110001110000	Food production
*Natrinema thermotolerans* A29	2914868299	110001110000	Food production
*Natrinema thermotolerans* DSM 11552	2534681901	110001110000	Aquatic
*Natronomonas* sp. LN261	3001203943	000001100001	Terrestrial
*Salinilacihabitans rarus* AD-4	8054413294	110001110001	Aquatic

^1^ Identifier for genomes in the Integrated Microbial Genomes & Microbiomes website. ^2^ The 12-digit binary number encodes the presence (“1”) or absence (“0”) of denitrifying enzymes in the following order narG, narH, narI, napA, napB, nirK, nirS, norB, norC, norV, norW, and nosZ.

## Data Availability

Files for datasets and visual analytics resource are available on the at https://github.com/qeubic/denitrification (accessed on 23 March 2024). The list of genomes annotated with the KEGG Orthology (KO) term identifier (e.g., “K00376” for nitrous-oxide reductase nosZ) can be retrieved from Integrated Microbial Genomes and Microbiomes (IMG/M) using the following webpage uniform resource locator: https://img.jgi.doe.gov/cgi-bin/m/main.cgi?section=FindFunctions&page=findkogenomelist&ko_id=KO:K00376&taxonChoice=allIsolates&data_type. The twelve KO identifiers are K00370, K00371, K00374, K02567, K02568, K00368, K15864, K04561, K02305, K12264, K12265, and K003763.

## Supplementary Materials

The following supporting information can be downloaded at https://www.mdpi.com/article/10.3390/microorganisms12040791/s1: Text S1: Information on content of the spreadsheet file, Table S1: Categories of the dataset columns, (2) Table S2: Microbial denitrification potential dataset; and (3) Figure S1: Visual of the microTrait framework. The interactive versions of the figures, worksheets and dashboards are available at https://public.tableau.com/app/profile/qeubic/viz/microbial_denitrifiers/abstract. The visual analytics file can also be downloaded and used as offline software using the free Tableau Reader (https://www.tableau.com/products/reader/download).

## Appendix A

The rules for categorizing the denitrification potential are based on the enzymes involved in the canonical denitrification steps [10] (Figure A1). The genes for non-enzyme proteins such as regulatory proteins are not included. Thus, the actual functional performance of a microbial strain in an ecological setting is influenced by other factors such as genetic, physiological, and environmental factors [62,97].

## Appendix B

The results of a search in Google Scholar with the search text (“absence of nosZ” denitrification), a search for articles documenting incomplete denitrification, included an open-access article published in 2022 by Jaio et al. [63]. The retrieved article included the categorization of 23 *Thermus* strains according to the presence in their genomes of eight genes encoding enzymes in the denitrification pathway. A comparison of the findings from this report and Jaio et al. shows agreement in the categorizations including for eight strains that do not have genes for the denitrification enzymes (Figure A2).

## Appendix C

We used microTrait’s denitrification rules [10] to assign 866 archaeal genomes retrieved from the IMG/M database [15] into one of the fifty-two denitrifying enzymes patterns and nine categories of the denitrification patterns (Figure A3).
